# Association between obesity and dental caries among adolescents in UAE: a pilot cross sectional study

**DOI:** 10.3389/froh.2023.1160428

**Published:** 2023-06-27

**Authors:** Manal Awad, Wegdan Bani Issa, Hadia Radwan, Randa Fakhry, Nabeel Al-Yateem, Rachel Rossiter

**Affiliations:** ^1^ Research Institute for Medical and Health Sciences, University of Sharjah, Sharjah, United Arab Emirates; ^2^ Department of Preventive & Restorative Dentistry, College of Dental Medicine, University of Sharjah, Sharjah, United Arab Emirates; ^3^ Department of Nursing, College of Health Sciences, University of Sharjah, Sharjah, United Arab Emirates; ^4^ Department of Clinical Nutrition and Dietetics, College of Health Sciences, University of Sharjah, Sharjah, United Arab Emirates; ^5^ School of Nursing, Paramedicine and Healthcare Sciences, Faculty of Science and Health, Charles Sturt University, Wagga Wagga, NSW, Australia

**Keywords:** dental caries, obesity, BMI—body mass index, waist circumference, cross sectional study, adolescents

## Abstract

**Background:**

Obesity and dental caries among adolescents is a growing worldwide public health issue. They share some common and modifiable influences. The objective of this study was to evaluate the association between obesity and dental caries among adolescents in the United Arab Emirates (UAE).

**Methods:**

This pilot cross-sectional study enrolled 161 adolescents 13–19 years old from private and public schools in the UAE. Participants were classified as normal weight, underweight, overweight or obese. Dental caries was diagnosed according to the criteria recommended by the World Health Organization (WHO). Independent *t*-tests were used to compare average number of decayed, missing and filled surfaces (DMFS) by age, sex, school type, mothers’ employment, BMI categories, waist circumference, oral health habits and plaque index. Additionally, a multiple linear regression model was applied to analyze the association between BMI, waist circumference and dental caries, adjusted for confounding factors considered in this study.

**Results:**

The average age of the participants was 16.2 ± 1.4 years old. The prevalence of overweight/obesity was 42% (*N* = 68) measured by BMI. In addition, 82% (*N* = 132) had average waist circumference and 18% (*N* = 29) with above average waist circumference. Overall, the average DMFS score was 4.35 ± 4.5, with significantly lower dental caries rates among girls ([3.3 (SD:4.0)] than boys (6.7 (SD:5.3), (*p* < 0.05). The linear regression model revealed that, being a male, attending a public school and having average waist circumference were all positively and significantly associated with dental caries (*p* < 0.05).

**Conclusion:**

Obesity measured by waist circumference was significantly associated with dental caries among adolescents in the UAE. Further research is required to investigate the complex association between obesity and dental caries and how dietary habits, oral hygiene habits, and parental socioeconomic status mediate the association between obesity and dental caries.

## Introduction

Dental caries remains one of the most prevalent chronic diseases worldwide (1–3). However, previous reports suggest that the profile of dental caries is heterogeneous across developing and developed countries, with large disparities reported between and within groups (3, 4). In 2017, dental caries affected 621 million children worldwide (3). Furthermore, in 2010, it has been estimated that 298 billion USD was spent on direct costs associated with dental caries, accounting for a substantial percentage of the total financial global expenditure (3). A recent systematic review in the MENA region (2) showed that there is a relatively high prevalence of dental caries in most MENA countries. For example, in Saudi Arabia, studies published from 2008 to 2018 showed a prevalence that ranged from 49%–91% (5). In the UAE, the prevalence of dental caries among school age children 11–17 years old was reported to be 75% and the mean DMFT score was 3.19 (SD:2.9) (2).

Dental caries is strongly influenced by sugar intake from energy-rich foods and drinks, which also play an important role in the development of obesity. Despite the number of studies conducted on this topic (6–16), the association between BMI and dental caries is still not clear (10, 12–23). Some studies demonstrated a positive association between BMI and dental caries (12–15), while others reported an inverse relationship (16–21) or no relationship (22–24). Findings from systematic reviews were also inconclusive (7, 12, 24–26). For example, based on their systematic review, Manohar et al. (26) concluded that there was a positive association between dental caries and obesity/overweight and that both conditions were associated with parental lower income and level of education. Hooley et al. (27) showed that almost half of the studies included in their systematic review reported no association between dental caries and BMI, as well as negative and positive associations between the two factors. They argued that this finding could be attributed to several factors including socioeconomic characteristics. Given the evidence from this systematic review, as well as, other studies (6, 10), it is possible that the association between obesity and dental caries is culturally dependent. For example, in some countries, higher rates of dental caries are observed among children of lower socioeconomic status (28). While in other countries the reverse is observed, and children from high socioeconomic groups eat more fermentable carbohydrates and may be at an increased risk of dental caries (29).

Dental caries and obesity are two conditions that could be associated with long term negative health consequences such as cardiovascular diseases and diabetes (30, 31). Furthermore, reports from previous studies showed that both diseases are directly linked to poorer quality of life among adolescents (32–34). Therefore, implementation of effective public health measures to address both conditions necessitates a better understanding of the nature of the association, in the context in which tailored interventions will be applied.

Arab adolescents exhibit different dietary habits and lifestyle factors compared to other countries and the prevalence of both obesity and dental caries is relatively high (35–37). In a recent study, it was reported that the prevalence of overweight and obesity among adolescents in the UAE was 34% (35). Moreover, Khadir et al. study (37) demonstrated that among 11–17 year old school children, 72% had at least one decayed tooth. However, information about the association between the two conditions is scarce. Therefore, the aim of the present study was to assess the association between obesity and dental caries among adolescents in the UAE.

## Subjects and methods

### Study design and setting

Data collected in the present study is part of a larger cross-sectional study that reported the prevalence of overweight/obesity among adolescents in the UAE (34). In the study, a one-stage cluster sampling technique was utilized and a sample of 932 adolescents aged 13–19 years old was recruited from intermediate and high schools (secondary education). Schools were randomly selected from lists obtained from the Ministry of Education and other private/public school governing bodies in the UAE's seven emirates (Sharjah, Dubai, Abu Dhabi, Ajman, Ras Al Khaimah, Fujairah, and Umm Al Quwain). The data used in this study comprised a sample of 161 adolescents who participated in the original cross-sectional study. To increase cooperation of the schools’ staff in data collection, and reduce interruptions to educational activities, principals of the selected schools were asked to choose the classes from grades 9–12 that would be available to participate in the study. All students in the selected classrooms were invited to take part in this research.

Participants in this study were male and female students in grades 9–12 who attended private or public schools, of any nationality and whose parents consented for them to take part in the study. Because management of chronic conditions requires following certain dietary and other life practices which may affect anthropometric measurements, students were excluded if they had any chronic condition, such as diabetes, cancer and mental conditions.

### Data collection

Participating students completed a self-report questionnaire that included questions about sociodemographic characteristics, frequency of tooth brushing and number of visits to the dentist. Trained research assistants administered the questionnaire in the selected classrooms. Participating students were then directed to the on-site school clinic where anthropometric measurements were taken by the same research team using standardized techniques. This was done to maintain consistency and uniformity in the data collection process across different sites. Two calibrated dentists conducted the oral examinations. Data were collected from September 2018 to May 2019, which was a regular school period excluding the summer holiday. In the UAE, there are no large seasonal variations during this part of the year and the temperature falls to an acceptable range; therefore, we anticipated no seasonal differences in anthropometric measurements.

### Measurements

The self-reported questionnaire included sociodemographic data (age, sex, nationality: nationals vs. non-nationals), and type of school (private vs. public) and mothers’ employment (employed/unemployed). Participants were also asked about the frequency of visits to the dentist (once a year or less than once a year) and frequency of tooth brushing (2–3 times/day or less than once/day).

#### Anthropometric measurements and indices

To ensure study reliability, a training and calibration process was carried out by one of the research team members who is an expert on anthropometric measurements (HR). Anthropometric indices included height (cm), weight (kg) and waist circumference (WC). For these measurements, students removed their shoes and wore minimal light-weight clothing as per World Health Organization (WHO) guidelines (37). Participants’ heights (to the nearest 0.1 cm) and weights (to the nearest 0.1 kg) were measured using a telescopic measuring rod (Seca 220) for column scales. Measurement for WC was recorded for each participant using an inextensible anthropometric tape (Seca 201). These measurements were taken while participants stood erect with their arms by their sides and feet close together. BMI (weight in kg divided by height in m^2^) was calculated and classified according to WHO criteria: underweight = BMI <3rd percentile; normal weight = BMI between the 3rd and 85th percentile; overweight = BMI between the 85th and 97th percentile; and obesity = BMI >97th percentile (37–39). However, because there are clinical limitations in using BMI to estimate obesity in adolescent populations and due to the lack of a gold standard to define obesity, in this study, we also reported WC as an obesity index. In the present study, waist circumference was categorized as average or above average using cutoff points for Arab adolescents (40).

### Oral health measurements

Oral examination was performed in the school premises using a portable dental chair and each student was comfortably seated. The dentists assessed oral hygiene status on a one-to-one basis using a disposable dental mirror with a light-emitting diode (LED) light and ball-end Community Periodontal Index (CPI) probe. Food debris was gently removed to avoid the under-recording of dental caries.

The dental caries activities in the participants were expressed as number of decayed, missing and filled teeth (DMFS). In this study, dental caries was recorded following the WHO recommendation (41). A tooth is recorded as missing due to caries if there was a history of extraction because of pain and or the presence of a cavity prior to extraction. Prevalence of plaque was assessed using the modified Quigley-Hein plaque index, in which, 0 = no plaque and 5 = plaque covering two-thirds or more of the crown of the tooth. Students lined up in a classroom and all examinations were conducted by two trained dentists who were calibrated for diagnosing decayed teeth and the plaque index. The degree of agreement was assessed, and the kappa coefficients were more than 0.80.

Dental caries experience was measured via the decayed–missing–filled surface (DMFS) index. DMFS is a cumulative measurement obtained by summing the number of decayed (D), missing (M), and filled (F) surfaces. A tooth surface that is decayed but has not been filled would be recorded as decayed (D). When a tooth was extracted due to caries, it would be recorded as missing (M). A tooth was recorded as filled (F) when it was permanently filled without caries.

### Data analysis

We used SPSS version 26 for all statistical analyses. Descriptive characteristics were calculated (frequency, percentage, mean, and standard deviation). Next, we used bivariate analysis to assess the differences in categorical variables and DMFS scores using independent *t*-test and analysis of variance (ANOVA).

A multilevel regression model was performed to take into account the schools cluster. In the first phase a “null model” without any independent variables was analyzed to verify whether there was a significant variation across the clusters (schools) regarding DMFS scores. The “null model” indicated that there was no significant clustering effects and all of the observations in the data can be treated as independent using single level model.

Therefore, Linear multiple regression was utilized to assess the association between dental caries (DMFS) (the dependent variable) and obesity (BMI and WC) (the independent variable) adjusted for sociodemographic factors, school, and plaque index. Results of the linear regression model using alpha level 0.05 (two-sided) was considered statistically significant.

### Ethics

Ethical approval to conduct this study was obtained from the University of Sharjah Research Ethics Committee (REC/15/12/10) and the UAE Ministry of Health and Prevention (MOHAP REC-11). Parental consent was obtained for each student that agreed to participate.

## Results

### Participants’ characteristics, dental caries activities and oral health habits

Table 1 presents the participants’ characteristics. The mean age of the participants was 16.2 (SD: 1.4). In this study 109 (67.7%) attended public schools, and 41 (25%) children indicated that their mothers were employed. The mean DMFS scores was 4.35 ± 4.5. The prevalence rate of overweight/obesity was 42% (*N* *= *68) (BMI ≥85th percentile).

**Table 1:** Characteristics of participating adolescents (*n = *161).

Characteristics	Frequency (%) or mean ± SD
**Gender**	
Male	30 (18.6)
Female	131 (81.4)
Age (years)	16.2 ± 1.4
**Nationality**	
UAE National	116 (72)
Non-UAE National	45 (28)
**Type of school**	
Public	109 (67.7)
Private	52 (32.3)
**Mother's employment**	
Employed	41 (25)
Unemployed	120 (75)
**BMI**	
Underweight/Normal	93 (58)
Overweight/Obese	68 (42)
**Waist circumference**	
Average	132 (82)
Above average	29 (18)
**Frequency of tooth brushing**	
2–3 times/day	111 (68.9)
Once a day or less	50 (31.1)
**Frequency of dental visits**	
Once a year or more	101 (62.7)
Less than once a year	60 (37.3)
DMFS	4.35 ± 4.5
D	0.99 ± 1.8
M	0.28 ± 0.7
F	1.8 ± 1.7

DMFS, number of decayed, missing, filled teeth; BMI, body mass index.

Table 2 shows the associations between sociodemographic factors, school, and obesity-related indices by DMFS total and separate component scores. UAE nationals had significantly higher DMFS scores and higher D scores compared to non-nationals (*p* < 0.05). In addition, children who attended private schools had significantly lower DMFS scores compared to children in public schools ((mean DMFS: 1.38 (SD: 1.9) and 5.2 (SD: 4.7), respectively)). Frequency of teeth brushing, visits to the dentist and BMI were not associated with dental caries activities.

**Table 2:** DMFS scores according to factors considered in the study (*n = *161).

Variable (*N*)	DMFS Mean (SD)	Decay Mean (SD)	Missing Mean (SD)	Filled Mean (SD)
**Gender**				
Male (30)	6.7 (5.3)*	2.3 (2.2)*	0.57 (0.9)*	1.3 (1.5)
Female (131)	3.3 (4.0)	1.2 (2.1)	0.19 (0.6)	1.1 (1.7)
**Nationality**				
UAE National (116)	4.3 (4.5)*	1.7 (1.9)*	0.28 (0.7)	1.1 (1.6)
Non-UAE National (45)	3.2 (4.3)	0.6 (1.6)	0.22 (0.06)	1.3 (1.8)
**Schools**				
Private (52)	1.38 (1.9)*	0*	0.17 (0.3)*	0.52 (0.9)*
Public (109)	5.2 (4.7)	2,1 (2.4)	0.32 (0.78)	1.46 (1.9)
**Mother's employment**				
Employed (41)	3.3 (3.0)	1.4 (2.5)	0.31 (0.8)	1.2 (1.7)
Unemployed (120)	4.2 (4.8)	1.3 (2.1)	0.12 (0.3)	1.1 (1.6)
**Visit to the dentist**				
Once a year or more (101)	3.9 (4.7)	1.3 (2.0)	0.3 (0.8)	1.5 (0.14)
Less than once a year (60)	3.8 (4.0)	1.7 (2.4)	0.2 (0.5)	1.9 (0.24)
**Frequency of brushing**				
2–3 times/day 111	3.8 (4.7)	1.2 (2.0)	0.27 (0.8)	1.5 (0.2)
Once a day or less 50	4.3 (3.6)	1.8 (2.5)	0.24 (0.5)	1.9 (0.3)
**BMI**				
Normal (93)	4.0 (4.8)	1.5 (2.3)	0.3 (0.7)	0.9 (1.6)
Overweight/Obese (68)	3.9 (3.8)	1.3 (1.9)	0.20 (0.6)	1.4 (1.7)
**Waist circumference**				
Average (132)	3.98 (4.80)	1.82 (1.76)	0.3 (0.74)	1.14 (1.64)
Above average (29)	3.83 (2.01)	1.32 (2.3)	0.10 (0.3)	1.21 (1.8)

DMFS, number of decayed, missing, filled teeth; BMI, body mass index.

* *p*-value < 0.05.

The results of the multiple linear regression (Table 3) show that females had significantly lower DMFS scores compared to males (*B *= −2.71, 95% CI: −4.50, −0.91). Having above average waist circumference was also associated with significantly lower DMFS scores (*B *= −2.11, 95%CI: −4.05,0.0). Higher plaque index was associated with significantly higher DMFS scores (*B *= 3.02, 95% CI: 0.85, 5.2). In the regression model, BMI was not associated with DMFS scores.

**Table 3:** Linear regression analysis for the association between DMFS and oral health habits and BMI.

Variables	B	95% Confidence intervals	
Male*	1		
Female	−2.71	−4.50	−0.91
Age	0.19	−0.42	0.82
**Nationality**			
UAE National*	1		
Non-National	0.61	−0.94	2.133
**School**			
Private*	1		
Public	2.11	−0.23	4.67
**Mother employed**			
No*	1		
Yes	−0.41	−1.90	0.99
**BMI**			
Average*	1		
Overweight/Obese	0.36	−1.14	1.9
**Waist circumference**			
Average*	1		
Above average	−2.01	−4.05	0.0
**Brushing teeth**			
2–3 times/day*	1		
Once/day or less	−0.06	−1.61	1.17
Visit a dentist			
Once a year or more*	1		
Less than once a year	−0.22	−1.55	1.06
Plaque index	3.02	0.85	5.20

DMFS, number of decayed, missing, filled teeth; BMI: body mass index.

* Reference category.

** *p* < 0.05.

## Discussion

The main objective of this study was to assess the association between obesity and dental caries among adolescents in the UAE. The chief finding of the present pilot study is that among adolescents, obesity measured by BMI was not associated with dental caries activities. To the contrary, our findings show that adolescents with above average waist circumference had significantly less tooth decay that those with average waist circumference**.** This relationship was persistent after adjusting for age, sex, type of school, mother’s employment status, oral health habits and plaque index. This significant association is in agreement with the findings reported by Yang et al. (20), in which data from their study among Chinese children suggested that overweight children had lower rates of tooth decay. Similar findings were also reported in the Philippines among 1951 children aged 11–13 years, in which, there was a significant association between dental caries and below average BMI (42). One possible explanation is that sedentary lifestyle and lack of exercise among adolescents are important contributors to obesity, since these two factors are not directly related to dental caries, an inverse relationship between obesity and dental caries is possible. Another explanation is that malnutrition can cause enamel hypoplasia, salivary glandular hypofunction, and salivary compositional changes which lead to the development of tooth decay (17).

To the contrary, findings from Norway (43), and Saudi Arabia (44) showed that above average waist circumference was significantly associated with increase in dental caries among adolescents. It has been suggested that waist circumference is a more accurate anthropometric measure of central adiposity and more sensitive than BMI. WC probably reflects visceral and subcutaneous fat and hence total fatness, while BMI does not distinguish between fat and fat–free masses. This may explain the significant association with dental caries (43, 44). Nevertheless, we used waist circumference to assess obesity and our findings support a negative association between above average waist circumference and dental caries.

Assessing the relationship between obesity and dental caries is challenging, because findings from our study, as well as previous studies (6–9, 12, 24, 26, 30, 43–45), raise several questions regarding our understanding of the association between obesity and dental caries. It is well known that dental caries is mainly caused by sugar consumption, while obesity is not specifically related to sugar consumption, but to the consumption of a diet that is high in fat and consumption of large portion sizes (24, 46). Accordingly, it is possible that obese adolescents maintain good oral hygiene reflected in lower caries activities, while also consuming energy-dense diets (24). Therefore, the lack of association between dental caries and obesity would be understandable. However, given our findings, as well as that of other researchers (6–9, 10, 23, 43), it appears that the association between body weight and dental caries is complex. Rather than assuming a causal pathway, the possibility that the two conditions share common risk factors, such as diet and socioeconomic status should not be ruled out (6, 10). Common risk factors make studies that addressed the association between obesity and dental caries relevant from a public health perspective, in the development of preventive measures that target both health problems in a cost-effective manner (47, 48).

Despite the lack of an association between frequency of tooth brushing and number of visits to the dentist with DMFS scores observed in this study, the positive significant association between plaque scores and dental caries suggests poor oral hygiene practices, leading to the retention of dental plaque, the increase in streptococcus mutans colonization and possible loss of enamel.

Interestingly, females in our study had significantly lower prevalence of tooth decay than males. Usually, females are expected to exhibit a higher caries rate due to earlier tooth eruption, and thus longer exposure to cariogenic processes (49). However, sex related behaviors also likely contribute to differences in caries. It is also possible that in some cultures boys often have more access to sweets and calorie rich foods than girls (6), along with girls’ desire to stay slim, could contribute to the reduction in consumption of sugar and consequently reflected in lower prevalence of tooth decay.

Overall, adolescents in this study had relatively high prevalence of dental caries compared to their counterparts from Norway (43), Egypt (50) and China (51). Protecting permanent teeth from tooth decay is critical to reduce negative psychological, health and well-being consequences. Therefore, oral and dental health education and promotion should be more comprehensively integrated into adolescents’ schools. Our findings confirm previous studies in the UAE (36, 51–53), that showed significantly higher DMFS scores among students in public schools. In this study participants from private schools did not have any decayed teeth, indicating that being in private schools maybe associated with better utilization of dental services and better oral hygiene practices. These findings call for necessary public health initiatives to reduce the relatively high incidence rate of dental caries that is repeatedly reported in public schools in the UAE.

### Strengths and limitations

Our findings are the first to assess the association between obesity and dental caries among adolescents in the UAE. We also considered multivariate models that took into account the contribution of several factors in addressing this relationship. Our results highlight the complexity of the association and the need for longitudinal studies to confirm or contest the relationship between obesity and dental caries. However, our study has certain methodologic limitations, including that this is a pilot study, and a larger sample size is needed to establish the association between obesity and dental caries in this population. We also did not include specific information about socioeconomic status of the study participants. However, school type is indicative of parental economic status in the UAE (54). Because this is a cross-sectional study, we cannot assume causality between obesity and dental caries. In addition, sugar intake was not assessed in this study. The majority of participants were females, and with more representation from public than private schools. Future studies may apply the International Caries Detection and Assessment System (55) to have a more comprehensive assessment of dental caries status among adolescents.

## Conclusions

To conclude, in the present study, waist circumference was significantly associated with dental caries. However, obesity and dental caries are both multifactorial; therefore, further research is needed to determine the direction and strength of the association. Our finding of the relatively high prevalence of dental caries among adolescents in the UAE underscores the importance of public health measures to increase oral health promotion efforts.

Finally, given the complexity of the association between obesity and dental caries, cohort studies are recommended to clarify the mechanisms of the association between BMI and dental caries.

## Data Availability

The original contributions presented in the study are included in the article, further inquiries can be directed to the corresponding author/s.
